# Undetectable or low (<1 ng/ml) postsurgical thyroglobulin values do not rule out metastases in early stage differentiated thyroid cancer patients

**DOI:** 10.18632/oncotarget.24766

**Published:** 2018-04-03

**Authors:** Alfredo Campennì, Luca Giovanella, Salvatore Antonio Pignata, Antonio Vento, Angela Alibrandi, Letterio Sturiale, Riccardo Laudicella, Alessio Danilo Comis, Rossella Filice, Giuseppe Giuffrida, Maria Elena Stipo, Salvatore Giovinazzo, Francesco Trimarchi, Rosaria Maddalena Ruggeri, Sergio Baldari

**Affiliations:** ^1^ Department of Biomedical and Dental Sciences and Morpho-Functional Imaging, Nuclear Medicine Unit, University of Messina, Messina, Italy; ^2^ Nuclear Medicine and PET/CT Centre, Oncology Institute of Southern Switzerland, Bellinzona, Switzerland; ^3^ Department of Clinical and Experimental Medicine, Unit of Endocrinology, University of Messina, Messina, Italy; ^4^ Department of Economical, Business and Environmental Sciences and Quantitative Methods, University of Messina, Messina, Italy; ^5^ Accademia Peloritana dei Pericolanti, University of Messina, Messina, Italy

**Keywords:** differentiated thyroid cancer, 131-radioiodine treatment, 131-radioiodine thyroid remnant ablation, thyroglobulin, post-therapy whole body scintigraphy

## Abstract

**Background:**

Differentiated thyroid cancer (DTC) work-up is based on (near)total-thyroidectomy plus thyroid remnant ablation (TRA) with 131-radioiodine in many patients, and long-life follow-up. ^131^I-post therapy whole body scan (pT-WBS) and serum thyroglobulin (Tg) are used in identifying metastatic patients. Some authors have evaluated the possibility of using post-surgical Tg (ps-Tg) values in deciding for or against TRA. The aim of our study was to verify the diagnostic accuracy of ^131^I-pT-WBS and SPECT/CT imaging (post-therapeutic imaging) compared to serum Tg levels in detecting metastases in early stage of DTC patients.

**Results:**

Post-therapeutic imaging revealed metastases in 82 out of 570 (14.4%) patients. Metastases were successively confirmed by other diagnostic tools or by histology (sensitivity and PPV = 100%). Seventy-three out of 82 patients (90.2%) showed ps-Tg levels ≤1 ng/ml. In fifty-four per cent of patients, serum Tg levels at TRA remained ≤1 ng/ml.

**Conclusion:**

In conclusion, ps-Tg levels cannot be used in deciding for or against TRA. In early stage of DTC, post-therapeutic imaging (^131^I-pT-WBS and SPECT/CT) is an accurate method of detecting metastases, also in patients with stimulated serum Tg values ≤1 ng/ml

**Methods:**

We retrospectively reviewed the records of 570 consecutive patients affected by pT1-pT3 DTC (F = 450, M = 120), referred to our Nuclear Medicine Units in the last five years to perform TRA after (near)-total-thyroidectomy.All patients underwent TRA 3-4 months after thyroid surgery either in euthyroid or in hypothyroid state. Serum Tg values evaluated in post-surgical period and at TRA were matched with post-therapeutic imaging results.

## INTRODUCTION

Thyroid cancers occur in 2-5% of thyroid nodules, with an incidence of 3.8% of all new cancer diagnosis in 2014 [1, 2]. The most frequent form is differentiated thyroid cancer (DTC), while other types are very rare but more aggressive [3–5].

DTC incidence has been increasing in the last few decades, with a large prevalence of small tumours (≤ 2 cm), which can be referred to a wider use of diagnostic methods, such as ultrasound (US) and fine-needle ago-cytology (FNAC) [6].

The work-up of DTC patients was traditionally based on (near)-total thyroidectomy [(n)TT] plus radioiodine (RAI) thyroid remnant ablation (TRA) in most patients, and long-life follow-up.

According to the latest American Thyroid Association (ATA) guidelines, the role of TRA has been revised. TRA is clearly indicated in high risk patients while it is not indicated in low risk patients and discouraged in many intermediate risk cases.

^131^I-post therapy whole body scan (pT-WBS), with or without SPECT/CT imaging (post-therapeutic imaging) and serum thyroglobulin (Tg) measurements, are used in identifying metastatic patients. In addition, in the recent past, some authors have evaluated the possibility of using post-surgical Tg (ps-Tg) values (both in L-T4 therapy and/or after TSH-stimulation) in deciding for or against TRA [7–11].

However, serum Tg values may be undetectable or low (i.e. ≤ 1 ng/ml) both in the post-surgical period and at the time of TRA (a-Tg), also in patients with loco-regional and/or distant metastases detected by post-therapeutic imaging and complementary diagnostic tools [12–14].

The aim of the present study was to verify the diagnostic accuracy of serum Tg values in a large cohort of patients, obtained in the post-surgical period and at the time of TRA, compared to post-therapeutic imaging, in identifying metastases in early stage DTC patients.

## RESULTS

**Figure 1:**
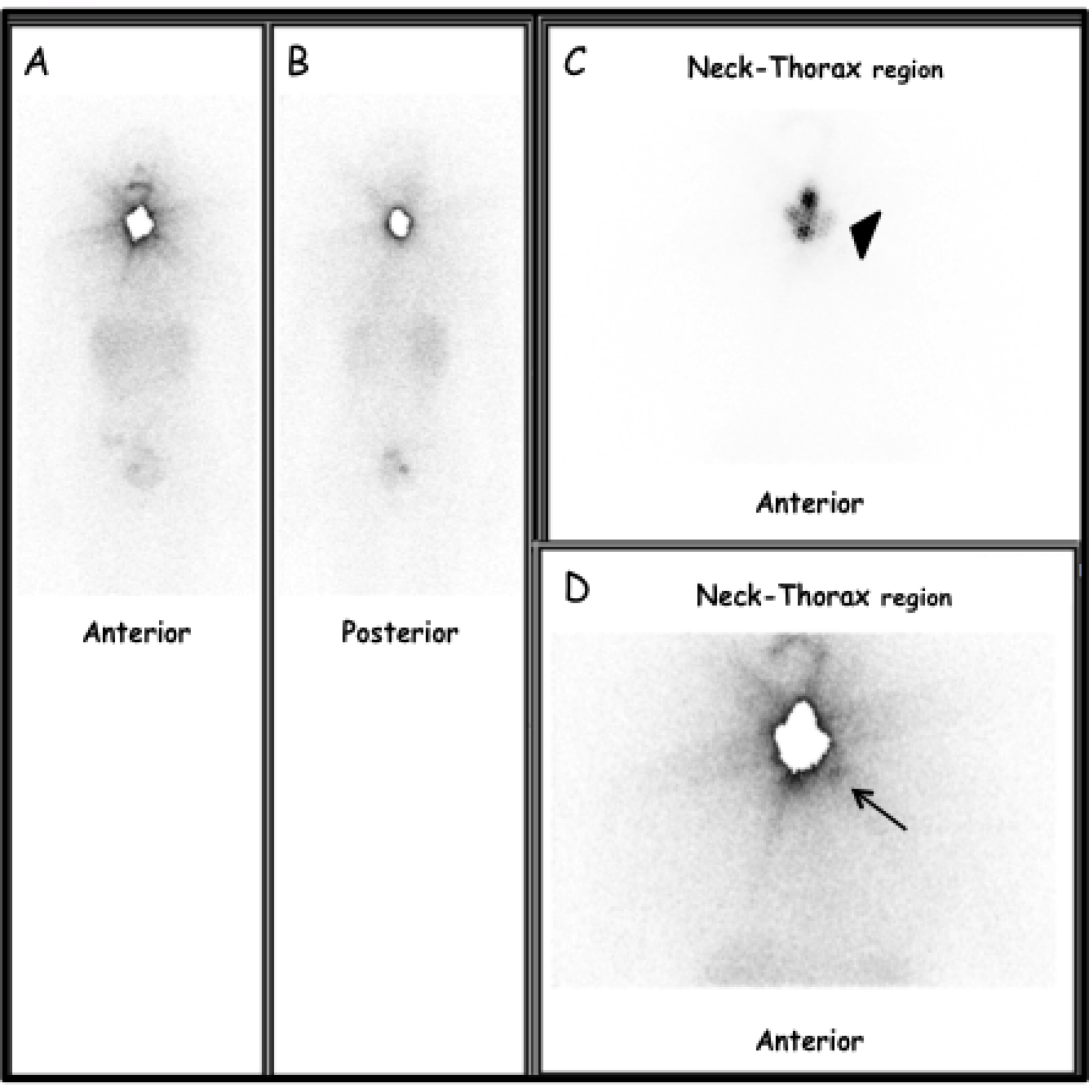
Thirty-seven-year-old woman, pT1bNxMx-PTC (classic variant) located in the left lobe (**A**) Anterior view and (**B**) posterior view pT-WBS. Static images of the neck and thorax regions in Anterior (**C**) and Posterior (**D**) views obtained 5 days after TRA (2220 MBq after rhTSH stimulation). Moderate to intense radioiodine uptake (black arrow-head) in thyroid bed (i.e. thyroid remnant). Focal area of slight radioiodine uptake, corresponding to left lateral lymph node metastasis (black arrow). At TRA, serum TSH, Tg-Ab and Tg levels were 141 µIU/ml, < 4 ng/ml and 1.0 ng/ml, respectively. Lymph-node metastasis was successively confirmed by targeted nUS and fine needle aspiration cytology (FNAC) and Tg measurement in the aspirate fluid (> 500 ng/ml).

**Figure 2:**
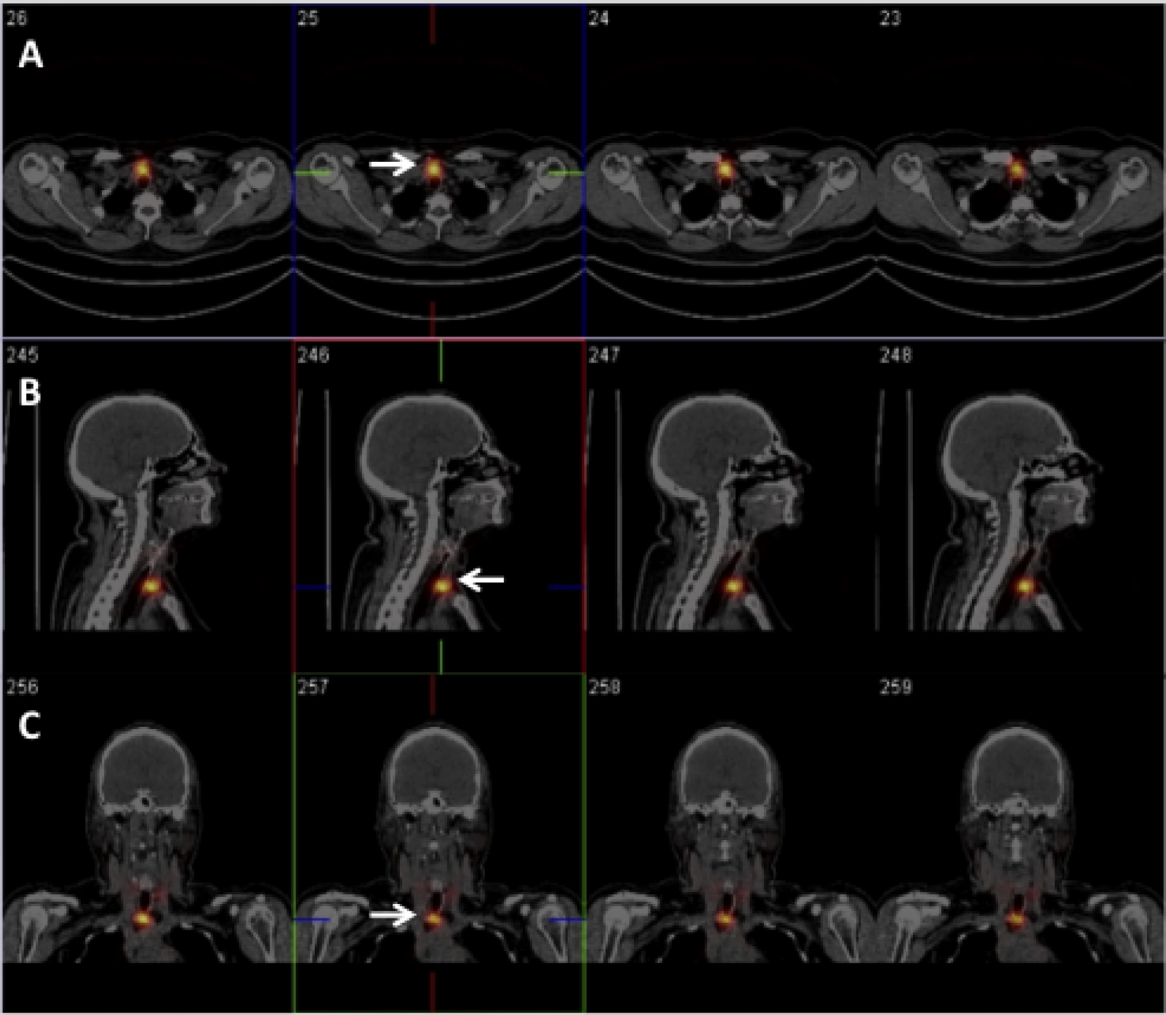
Fifty-eight-year-old woman, pT1aNxMx-PTC (follicular variant) located in the isthmus Patient underwent TRA after 5-weeks L-thyroxine withdrawal. RAIU: 2.5%. Five days after TRA (2753 MBq), axial (**A**), sagittal (**B**) and coronal (**C**) SPECT/CT images identified a focal area of quite intense radioiodine uptake corresponding to central compartment lymph node metastasis (white arrow). At TRA, serum TSH, Tg-Ab and Tg levels were 70 µIU/ml, < 4 ng/ml and 1.0 ng/ml, respectively. Lymph-node metastasis was also confirmed by targeted nUS (10 mm in size).

RAI avid metastases were detected in 82 out of 570 (14.4%) patients (F = 60, M = 22; median age 49 years; range 17–82 years) by post-therapeutic imaging (Figures 1, 2).

In all patients, metastases were successively confirmed (sensitivity and PPV = 100% for both) by targeted morphological and/or morpho-metabolic studies [i.e. nUS, diagnostic CT (with and without contrast media agent), MR, ^18^F-FDG-PET/CT] and/or node biopsy with Tg measurement in the aspirate fluid [20 out of 82 metastatic patients (24%)] and/or histology [16 out of 82 patients (19.5%)].

No significant differences in terms of gender and age were observed between metastatic and non-metastatic patients (*p* = 0.792 and *p* = 0.467, respectively).

Seventy-five out of all metastatic patients (91.5%) were affected by PTC, while seven (8.5%) had FTC. Thirty-two patients were treated in euthyroid state (F = 22, M = 10 F/M ratio = 2.2:1, median age 48 years, range 17-82) while the remaining were treated in hypothyroid state (F = 38, M = 12, F/M ratio: 3.2:1, median age 50.5 years, range 25-73).

In metastatic patients, the median administrated radioiodine activity was 3700 MBq (range 1110-9250) without significant differences compared to non-metastatic patients (*p* = 0.650).

In patients treated in hypothyroid state, median RAIU value was 6.6% (range 2–29%). No significant differences in terms of RAIU values were observed between metastatic and non-metastatic patients (*p* = 0.074).

Finally, comparing TSH values evaluated at TRA (obtained either after L-T4 withdrawal or after rhTSH administration) of metastatic patients compared to non-metastatic patients, we did not observe any significant differences (*p* = 0.929)

Demographic, clinical, pathological and scintigraphic data of 82 metastatic patients are reported in Supplementary Table 1.

### Serum Tg values evaluated during post-surgical period (ps-Tg)

In 10 out of 82 metastatic patients, ps-Tg levels were higher than 1 ng/ml (median 4.39 ng/ml), but only three showed ps-Tg values suspicious for metastatic disease (18.7, 102 and 4700 ng/ml, respectively). None of these patients declared symptoms or presented signs that could be referred to loco-regional and/or distant metastases. In contrast, in 72 out of the 82 metastatic patients (88%), ps-Tg value was below compared to sensitivity cut-off (i.e. ≤ 0.15) in 61 patients, while in the remaining 11 patients ps-Tg ranged from 0.16 to 1 ng/ml.

Serum TSH values were < 1 µUI/ml (median: 0.178 µUI/ml, range 0.005-0.92) in all of these patients except in a female patient in whom the value was slightly higher than 1 (i.e. 1.3 µUI/ml).

### Serum Tg levels at TRA (a-Tg)

In 40 out of 72 metastatic patients (55.5%) who had a ps-Tg < 1 ng/ml, the serum a-Tg levels remained ≤1 ng/ml (Group A). In particular, 24 out of 72 patients (33%) also had a serum a-Tg value under the sensitivity cut-off (i.e. ≤ 0.15 ng/ml). Twenty-one of these patients were treated in euthyroid state (after rhTSH administration) while the remaining were treated in hypothyroid state.

In sixteen patients, serum a-Tg values ranged from 2.2 to 8.46 ng/ml (median 4.2 ng/ml) (Group B). In all these patients, ps-Tg were ≤ 1 ng/ml. Most of these patients (14 out of 16, or 87.5%) were treated in hypothyroid state.

Serum a-Tg values were ≥ 10 ng/ml (median 36 ng/ml, range 10.0-4.700) in 26 out of 82 patients (31.7%) only (Group C).

Even if hypothyroidism generally produces a stronger stimulus than rhTSH-administration on thyroid cells to release Tg, we did not observe significant differences between hypothyroid and euthyroid patients in terms of serum a-Tg levels (*p* = 0.653).

**Figure 3:**
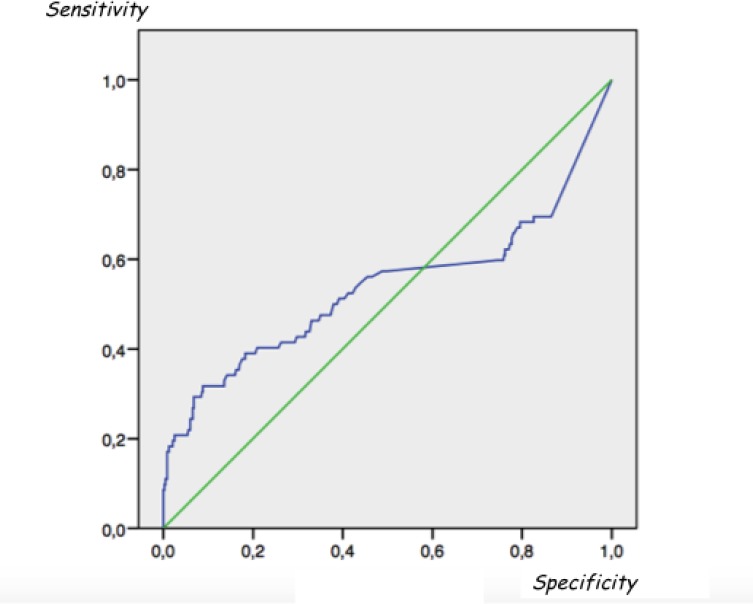
Receiver operating characteristic (ROC) analysis did not find any serum Tg cut-off level able to distinguish between metastatic and non-metastatic patients Area under the ROC curve (AUC) = 0.53.

Considering serum a-Tg values of all patients and performing a receiver operating characteristic (ROC) curve analysis, we did not find any serum Tg cut-off level able to distinguish between metastatic and non-metastatic patients (AUC = 0.53) (Figure 3). However, considering both all enrolled patients and serum a-Tg values ≥ 10 ng/ml as suspicious for metastatic disease, the overall sensitivity, specificity, accuracy, PPV and NPV of serum a-Tg values in detecting metastatic patients were 31.7%, 96%, 86.6%, 56.5% and 89.3%, respectively.

Taking this threshold value into account, the risk of having metastatic lesions in patients with serum a-Tg values ≥ 10 ng/ml is about 45% higher than in other patients (odds ratio = 4.53).

Finally, we observed a significant linear correlation between a-Tg values and lymph-node metastasis size (*p* <0.001).

### Serum Tg values *vs* post-therapeutic imaging results *vs* pT-staging

Considering A and B Groups together (*n* = 56), median serum a-Tg value was 0.56 ng/ml (range 0.14–8.46). Post-therapeutic imaging showed lymph-node or lung and lymph-node metastases in 55 (98.2%) and 1 (1.8%) patients, respectively. In 32 out of 56 patients, metastatic lymph-nodes were located at VI or VII Robbins’s level, while in the remaining patients they were located in lateral neck compartments. The size of metastatic lymph-nodes located in the neck central compartment was not significantly different compared to that of lymph-nodes located in the lateral-neck levels (*p* = 0.703). Interestingly, the patient with lymph-node and lung metastases had a pT1b primary lesion located in the isthmus, thus confirming this topography as a risk factor for metastatic disease development, as already reported [16]. In this patient, the involved lymph-node was located in the central compartment (VI level of Robbins).

Comparing Group A and B patients with respect to their pT-staging, 43, 3 and 10 had pT1 (a or b), pT2 and pT3 lesions, respectively. Interestingly, pT3 lesions were prevalent but not in a significant way among Group B patients with respect to Group A patients: 6 out of 16 (37.5%) vs 4 out of 40 (10%) (*p* = 0.105).

Considering Group C patients (n = 26), median serum a-Tg value was 14 ng/ml (range 10.0-4700). Post-therapeutic imaging showed lymph-node, lung, lung and lymph-node, bone, lung and bone metastases in 20 (77%), 2 (7.7%), 1 (3.8%), 2 (7.7%) and 1 (3.8%) patients, respectively. In patients with lymph-node metastases, post-therapeutic imaging showed at least two areas of abnormal radioiodine uptake (i.e. lymph-node metastases). In 13 out of 20 patients, metastatic lymph-nodes were located at VI or VII Robbins’s level while in the remaining patients they were located in lateral neck compartments. The size of metastatic lymph-nodes located in the neck central compartment was significantly different compared to that of metastatic lymph-nodes located in the lateral-neck levels (*p* = 0.007).

Comparing Group C patients with respect to their pT-staging, 13, 2 and 11 had pT1 (a or b), pT2 and pT3 lesions, respectively. Interestingly, pT3 lesions were prevalent among Group C patients with respect to Groups A and B patients: 11 out of 26 (42.3%) vs 10 out of 56 (17.9%) (*p* = 0.040).

All patients with ps-Tg >1 ng/ml were enclosed in this group. Half of them (5/10 or 50%) had a pT3 lesion while 4 patients had a pT1 lesion and only one a pT2 lesion.

Comparing Groups A and B with respect to Group C, we did not observe any significant differences in terms of gender, age and pT-staging (*p* = 0.155, *p* = 0.555 and *p* = 0.147, respectively). On the contrary, it is very important to note how the size of lymph-node metastases of Group C patients was significantly higher than that of Group A and B patients (*p* = 0.0003). The prevalence of distant metastases was significantly higher in Group C patients than Group A and B patients (23% *vs* 1.7%, *p* = 0.004).

Finally, taking into account all pT1a-stage cases only, we observed that 37 out of 226 (16.4%) patients had metastases at post-therapeutic imaging and 22 of them (59.4%) had a Tg < 1 ng/ml (Supplementary Table 2).

## DISCUSSION

To date, according to ATA guidelines [18], Tg measurement is an important tool in the management of DTC patients. In addition, some years ago, some Authors [7–9] reported on the possibility to use ps-Tg values in deciding for or against TRA.

However, during routine clinical observation, we noticed that in a not-negligible number of early stage DTC patients with negative Tg-Ab titers and undetectable or low serum Tg values (i.e. ≤ 1 ng/ml), evaluated both during post-surgical period and at TRA, post-therapeutic imaging showed radioiodine avid metastatic lesions.

Some authors have already reported on this topic [7, 12–14, 19–24] but in all except a manuscript published by Park *et al.* [12], the patient number was limited. In addition, in these papers, no information on tumour grading differentiation was reported and in all except Park’s paper, patients with positive Tg-Ab were included. Thus, in our study, we chose to rule out patients with possible interference factors to better evaluate the diagnostic accuracy of serum Tg values in detecting metastases in early stage of DTC patients.

Post-therapeutic imaging showed avid radioiodine metastases in 14.4% of our patients’ cohort. Interestingly, most of these patients (72/82, or 88%) had ps-Tg values ≤ 1 ng/ml and, in particular, in 61 of them (84.7%), ps-Tg was below sensitivity cut-off (i.e. ≤ 0.15 ng/ml). In addition, it is important to highlight that 44 out of 72 (61.1%) of these metastatic patients (56 out of all metastatic patients, 68.3%) were scored as pT1(a or b) according to pTNM classification [25]. Thus, these patients would not have undergone TRA according to ATA guidelines [18], also taking into account data already published on the possible role of ps-Tg levels in deciding for or against TRA [7–9].

On the contrary, our data show that ps-Tg values cannot be used in deciding for or against TRA, mainly in so-called low-risk patients that, to date, represent the largest number of DTC patients [26].

More in general, in early stage of DTC patients, mainly in so-called low-risk patients, serum Tg values, also obtained at TRA (i.e. a-Tg) cannot be useful in differentiating metastatic- from- non-metastatic patients and, in our series, no cut-off value was identified by ROC curve analysis. However, in the “clinical” evaluation of a-Tg, it is important to take into account the type of stimulation strategy (hypothyroidism *vs* rhTSH-stimulation) used to perform TRA since hypothyroidism may produce a stronger stimulus. However, in our series no significant differences were found among patients treated in hypothyroid or euthyroid state (*p* = 0.653).

Nevertheless, it is important to highlight how in our series, patients with a serum a-Tg level ≥ 10 ng/ml showed higher risk of having metastatic lesions than those with a serum a-Tg level <10 ng/ml (odds ratio = 4.53).

Also in our series, the risk of false negative serum Tg values was higher in patients with small loco-regional lymph-node metastases than in patients with either larger lymph-node or distant metastases, as already described [19, 24, 27, 28]. This is a significant problem, as, to date, most patients are affected by low risk cancers and a significant number of these patients have small loco-regional metastases rather than distant metastases [16, 29]. In this setting of patients, an accurate nUS performed by expert physicians can play an important diagnostic role in early detection of “suspected” lymph-nodes [30]. Thus, serum Tg measurement only would not have allowed to correctly identify a significant number of metastatic patients who, on the contrary, would have been wrongly considered as “disease free”.

Probably, these patients would not have had an increased risk of disease specific mortality [18] even if most were young-adult but it would have had negative effects on patients’ outcome, reducing quality of life [16].

Some reasons have already been proposed to explain serum Tg false negative results, such as the so-called “hook effect” [31] that may occur in the presence of high antigen levels. However, most of our metastatic patients had undetectable or low serum Tg values both during the post-surgical period and at TRA. Thus, this reason cannot explain our false negative results.

Other reasons have been reported [20], such as reduced Tg synthesis and/or release by metastatic cells or more rapid Tg clearance from the plasma. However, the first condition, as already reported [20] regards patients with marginally differentiated tumours who were ruled out from our series, while, unfortunately, we did not perform another test to rule out the last option (i.e. Tg may be cleared more rapidly from the plasma). However, more rapid Tg clearance from the plasma is such a very rare condition that Park and colleagues [12] performed another evaluation in their patients with false negative serum Tg, but the second test confirmed the first results.

Our data suggest that false negative serum Tg values may be due to small size of metastatic lesions, as already described [12, 32–34], coming from small neoplastic lesions (i.e. pT1). In our series, most metastatic patients had lymph-node metastases (77 out of 82 patients) and in 57% the median size of lymph-node metastasis was 7.5 mm. In the majority of these patients (53 out of 77 patients, or 68.8%), a primary neoplastic lesion was classified as pT1.

In addition, we think that both the number of lesion(s) and the type of tissue(s) involved with metastatic cells can play an important role in the reduced synthesis and/or in the reduced release of Tg into the blood.

In our metastatic patients with serum a-Tg values <10 ng/ml (Groups A and B) and lymph-node metastases, post-therapeutic imaging and targeted morphological studies showed few metastatic lesions of smaller size compared to patients with serum a-Tg values ≥ 10 ng/ml (Group C).

On the contrary, both patients with detectable ps-Tg and all patients with serum a-Tg values ≥ 10 ng/ml had a higher number of metastatic foci and/or larger size of lesions (lymph-node median size = 11 mm) and/or higher prevalence of distant metastases (i.e. lung and/or bone metastases) at post-therapeutic imaging than other patients (i.e. Groups A and B). Also in this setting of patients, nUS can be important both in early identification of metastatic lesions, thus improving/changing clinical management (e.g. performing lymphadenectomy before TRA) and in serving as a guide to perform an accurate node biopsy.

Thus, taking into account both our results and data already published [12], post-therapeutic imaging (i.e. pT-WBS and SPECT/CT) represents a very sensitive tool in early stage of DTC patients that allows us to correctly score the clinical risk of patients having metastatic lesions, taking into account that it may be different by pathological staging, as already described [35].

In order to reduce the healthcare costs of TRA and post-therapeutic imaging, considering both ATA guidelines and the small (but not negligible) number of patients with false negative serum Tg and positive post-therapeutic imaging, it might be useful to use 123-radioiodine diagnostic scintigraphy in early identification of patients with unsuspected metastases. However, to date, its use is very limited due to high cost.

In our work, there are two main limitations. The first limitation is represented by the study population which was mainly composed of PTC and only in very small part by FTC. Such a high prevalence of PTC is common in iodine-deficient countries, such as southern Italy and Switzerland. Patients from these countries are often affected by large goiters. As a consequence, the rate of patients with incomplete surgical resection may be higher than in other countries, thus limiting the specificity of a ps-Tg value greater than 1 ng/ml. The second limitation is represented by the fact that in our work, as in Park’s paper [12], metastatic disease was confirmed by node biopsy and/or histology in a small number of patients only.

## CONCLUSIONS

Our data demonstrate how ps-Tg values cannot be used in deciding for or against TRA and, of course, in predicting the outcome of patients, mainly in those classified as “low-risk”.

In early stage DTC patients, post-therapeutic imaging is significantly more accurate than serum Tg values in detecting DTC metastatic patients, also among those with a-Tg level ≤1 ng/ml.

## PATIENTS AND METHODS

### Patients

The records of 1102 DTC patients (F = 851, M = 251, median age = 45 years; range: 11-82 years; female to male ratio = 3.4:1) referred to our Nuclear Medicine Unit (at “G. Martino” University Hospital of Messina, Italy) or to the Department of Nuclear Medicine and Thyroid Centre, Oncology Institute of Southern Switzerland, Bellinzona/Lugano (Switzerland), from January 1, 2011 through December 31, 2014, were reviewed.

For the present study, we selected 570 out of 1102 patients affected by pT1N0(X)MX-pT3N0(X)MX DTC (F = 450, M = 120, median age = 49 years, range 17-82 years; female to male ratio 3.7:1).

Exclusion criteria were: 1) presence of loco-regional or distant metastases at the time of recruitment; 2) age ≤ 16 years; 3) positive thyroglobulin-antibody (Tg-Ab); 4) pT4 stage, 5) poorly-differentiated thyroid cancer.

Five hundred and sixty patients (98.2%) were affected by papillary thyroid carcinoma (PTC) while 10 (1.8) had follicular thyroid carcinoma (FTC).

Before thyroid remnant ablation (TRA), all patients underwent neck-ultrasonography (nUS), laboratory test (i.e. serum evaluation of TSH, FT3, FT4, Tg and Tg-Ab) obtained on L-T4 sub-suppressive therapy (two months after thyroid surgery: i.e. post-surgical period) and, those treated in hypothyroid state only, radioiodine thyroid uptake (RAIU), performed as already described [3]. In patients treated after rhTSH-stimulation, a-Tg was obtained three days after the last rhTSH-administration (thus two days after TRA).

Patients underwent TRA 3-4 months after thyroid surgery either in euthyroid state [by intra-muscular administration of recombinant human TSH (rhTSH) (*n* = 249, or 44%)] or in hypothyroid state (after 5-weeks of L-thyroxine withdrawal, *n* = 321, or 56%).

In all patients, a post-therapeutic imaging was acquired 5-7 days after TRA.

Finally, serum Tg values obtained both during the post-surgical period and at TRA were matched with post-therapeutic imaging results.

### Neck-ultrasonography

nUS was performed ≥ two months after surgery and before TRA by expert ultrasonographers. Studies were always performed with a high-resolution ultrasound system equipped with a high energy linear probe (14 MHz). Colour-Doppler study was also performed, if necessary. nUS evaluation included the thyroid bed and both central and lateral lymph-nodes stations. Patients with suspicious lymph-nodes (i.e. round shape, irregular borders, cystic appearance, hyperechoic punctuations and hypervascularization) underwent fine needle aspiration cytology (FNAC) and Tg measurement in the aspirate fluid. If the lymph-node was positive for malignancy, the patient underwent selective or radical lymphoadenectomy. These patients were not included in the present study. However, in this study, patients without lymph-nodes suspicious for malignancy were assessed.

### Laboratory test

Measurement of serum TSH (Chemiluminescent immunoassay, Beckman; normal values 0.3–4.2 mUI/L), Thyroglobulin (Tg) (immunoradiometric assay –IRMA- Cisbio; normal values 0–40), and anti-Thyroglobulin Antibodies (Tg-Ab) (Chemiluminescent immunoassay, Beckman; normal values 0-4) was obtained. Tg assay had a functional cut-off of 0.6 ng/ml and a sensitivity cut-off of 0.15 ng/mL.

### Radioiodine thyroid uptake (RAIU)

RAIU was evaluated 24 hours after Na-^131^I tracer activity (1.8 MBq) administration, as elsewhere described [15, 16].

### Radioiodine therapy (RIT)

Radioiodine activities administrated for TRA ranged from 1110 to 9250 MBq (median: 3700 MBq). Higher radioiodine activities were administered to patients with higher risk factors (e.g. aggressive variant of PTC, FTC, detectable ps-Tg).

### Post-therapeutic imaging [post-therapy whole body scan (pT-WBS) and SPECT/CT]

pT-WBS was obtained using a double-headed gamma camera (Millennium VG, GE Medical System. Chicago, Illinois, USA; Symens Symbia T-series. Munich, Germany) equipped with high-energy low-resolution parallel-hole collimators (HEHRPAR). In order to obtain a better target/background ratio, patients were required to drink at least 1.5 litres of water and take laxatives drugs some days before the study. The pT-WBS was integrated by single photon emission tomography-computed tomography (SPECT-CT) on request of attending nuclear medicine physician. When SPECT/CT images were obtained, nuclear medicine physicians verified if the abnormal radioiodine uptake corresponded to a defined morphological lesion (i.e. lymph-node, lung, bone or brain lesions).

### Post-therapeutic imaging evaluation

During retrospective analysis of the data, all post-therapeutic images were independently re-evaluated by two nuclear medicine physicians with more than twenty years of expertise, blinded to serum Tg levels. If they disagreed, they discussed and reached a consensus in all cases. Radioiodine uptake in the thyroid bed was considered as thyroid remnant while the radioiodine uptake located outside the thyroid bed and/or corresponding to lymph-node was considered as lymph-node metastasis.

### Other imaging studies

In patients with neck lymph-node metastases discovered by post-therapeutic imaging, a targeted nUS was also performed.

Contrast-enhanced CT and/or magnetic resonance (MR) and/or ^18^F-FDG-PET/CT studies were required in selected cases on indication of the attending physician.

In 60 out of 82 metastatic patients (73.2%), a ^99m^Tc-MIBI scan of the neck-thoracic regions was also obtained, as previously described [17].

In patients with lateral neck lymph-node metastases, an FNAC for cytological examination and Tg measurement in the aspirate fluid was also performed.

### Patients assessment

Patients with loco-regional and/or distant metastases at post-therapeutic imaging underwent surgery (whenever possible) and/or RIT with high radioiodine activity (5550-7400 MBq) 6-8 months after TRA.

Patients without loco-regional and/or distant metastases at post-therapeutic imaging were revaluated 6–12 months after TRA to verify the effectiveness of treatment by Tg measurements (both basal and after rhTSH-stimulation) and nUS. In patients with large thyroid remnant and/or undetectable serum Tg values at TRA, a diagnostic (185 MBq) radioiodine whole body scintigraphy was also obtained after rhTSH administration (standard protocol).

### Statistical analysis

Numerical data are expressed as median and categorical variables as number and percentage. Examined variables did not present normal distribution as verified by *Kolmogorov Smirnov test;* consequently, the non-parametric approach has been used.

*Mann Whitney test* was applied in order to evaluate the existence of statistically significant differences between patients with and without metastases, regarding age, RAIU, TSH and lymph-nodes size; the same test was used to assess differences between patients treated in euthyroid or in hypothyroid state.

*Chi Square test* was applied in order to assess differences in gender distribution in patients with and without metastases.

*Receiver Operating Characteristic (ROC)* curve was realized, to verify the possible optimal thyroglobulin cut-off value in differentiating between non-metastatic and metastatic patients, and the area under the curve (AUC) was calculated. Sensitivity, specificity, negative predictive value (NPV) and positive predictive value (PPV) of thyroglobulin were evaluated.

Statistical analyses were performed using SPSS 17.0 for Window package.

*P* < 0.050 two sided was considered to be statistically significant.

## SUPPLEMENTARY MATERIALS TABLES






