# Supplementary material for: Undetectable or low (<1 ng/ml) postsurgical thyroglobulin values do not rule out metastases in early stage differentiated thyroid cancer patients
Source: Oncotarget. 2018 Apr 3;9(25):17491–500. doi: 10.18632/oncotarget.24766 (PMC5915131; doi:10.18632/oncotarget.24766)
Supplement: Supplementary file 1 [file oncotarget-09-17491-s001.pdf]

# Undetectable or low (<1 ng/ml) postsurgical thyroglobulin values do not rule out metastases in early stage differentiated thyroid cancer patients

## SUPPLEMENTARY MATERIALS

**Supplementary Table 1: Demographic, clinical, pathological and scintigraphic data of 82 metastatic patients.** See Supplementary_Table_1

**Supplementary Table 2: Demographic, clinical, pathological and scintigraphic data of 22 pT1a patients with a-Tg <1 ng/ml and metastasis(es) at post-therapeutic imaging.** See Supplementary_Table_2
