# Supplementary material for: Undetectable or low (<1 ng/ml) postsurgical thyroglobulin values do not rule out metastases in early stage differentiated thyroid cancer patients
Source: Oncotarget. 2018 Apr 3;9(25):17491–500. doi: 10.18632/oncotarget.24766 (PMC5915131; doi:10.18632/oncotarget.24766)
Supplement: Supplementary file 2 [file oncotarget-09-17491-s002.doc]

**Supplementary Table 1: Demographic, clinical, pathological and scintigraphic data of 82 metastatic patients**

| **Patient Number** | **Age** | **Sex** | **Tumor site** | **Histology** | **pT-staging** | **Stimulation**  **strategy** | **RAIU**  **(%)** | **Activity (MBq)** | **Metastases topography on post-therapeutic imaging** | **Targeted Diagnostic studies** | **Lymph-nodes size** | **Node biopsy** | **Lympho-adenectomy** | **TSH**  **on L-T4**  **(uIU/ml)** | **ps-Tg**  **(ng/ml)** | **TSH**  **at TRA**  **(uIU/ml)** | **Tg**  **at TRA**  **(ng/ml)** |
| --- | --- | --- | --- | --- | --- | --- | --- | --- | --- | --- | --- | --- | --- | --- | --- | --- | --- |
|  |  |  |  |  |  |  |  |  |  |  |  |  |  |  |  |  |  |
| **1** | 17 | F | RL | PTC | pT1a | rhTSH | --------- | 3700 | LN (VI) | nUS | 8 mm | ------------------ | ----------------- | 0.27 | 0.37 | 157.3 | 7.60 |
| **2** | 26 | F | RL | PTC | pT1a | rhTSH | --------- | 4588 | LN (VI) | nUS | 7 mm | ------------------ | ---------------- | 0.03 | 0.24 | 75.2 | 0.35 |
| **3** | 34 | F | B | PTC | pT1a(m) | rhTSH | --------- | 2220 | LN (II) | nUS | 7 mm | ------------------ | ---------------- | 0.15 | 0.14 | 115.0 | 0.14 |
| **4** | 37 | F | LL | PTC | pT1b | rhTSH | --------- | 2220 | LN (III) | nUS | 8 mm | performed(*) | ---------------- | 1.30 | 0.14 | 141.0 | 1.00 |
| **5** | 40 | F | RL | PTC | pT1a | rhTSH | --------- | 4588 | LN (VII) | nUS-TC | 9 mm | ------------------ | ---------------- | 0.17 | 0.14 | 83.0 | 0.14 |
| **6** | 40 | F | LL | PTC | pT1a | rhTSH | --------- | 4588 | LN (VI) | nUS | 7 mm | ------------------ | ---------------- | 0.46 | 0.14 | 56.6 | 0.14 |
| **7** | 48 | F | LL | PTC | pT3 | rhTSH | --------- | 3700 | LN (VI) | nUS | 11 mm | ------------------ | performed(#) | 0.03 | 0.40 | 72.0 | 2.50 |
| **8** | 48 | F | B | PTC | pT1a(m) | rhTSH | --------- | 3700 | LN (VI) | nUs | 6 mm | ------------------ | ---------------- | 0.45 | 0.14 | 63.1 | 0.14 |
| **9** | 48 | F | B | PTC | pT1a(m) | rhTSH | --------- | 2948 | LN (I) | nUS | 6 mm | ------------------ | ---------------- | 0.01 | 0.14 | 145.9 | 0.14 |
| **10** | 53 | F | I | PTC | pT1b | rhTSH | --------- | 2220 | LN (VII) | nUS-CT | 11 mm | ------------------ | ---------------- | 0.24 | 0.14 | 102.1 | 0.63 |
| **11** | 53 | F | LL | PTC | pT1a | rhTSH | --------- | 2220 | LN (II) | nUS | 11 mm | performed(*) | performed(#) | 0.09 | 0.49 | 128.0 | 0.68 |
| **12** | 59 | F | RL | PTC | pT1a | rhTSH | --------- | 2220 | LN (IV) | nUS | 6 mm | ------------------ | ---------------- | 0.09 | 0.14 | 120.0 | 0.62 |
| **13** | 67 | F | B | PTC | pT2(m) | rhTSH | --------- | 3700 | LN (VI) | nUS | 6 mm | ------------------ | ---------------- | 0.30 | 0.14 | 167.0 | 0.14 |
| **14** | 68 | F | RL | PTC | pT1a | rhTSH | --------- | 3700 | LN (III) | nUS | 6 mm | ------------------ | ---------------- | 0.02 | 0.14 | 54.8 | 0.27 |
| **15** | 69 | F | LL | PTC | pT1a | rhTSH | --------- | 3700 | LN (II) | nUS | 9 mm | performed(*) | performed(#) | 0,00 | 0.14 | 141.0 | 0.31 |
| **16** | 25 | M | I | PTC | pT1a | rhTSH | --------- | 3700 | LN (VII) | nUS-CT-MR | 5 mm | ------------------ | ---------------- | 0.04 | 0.23 | 74.4 | 0.52 |
| **17** | 31 | M | B | PTC | pT1a(m) | rhTSH | --------- | 4588 | LN (III) | nUS | 7 mm | ------------------ | ---------------- | 0.42 | 0.14 | 88.8 | 0.14 |
| **18** | 43 | M | RL | PTC | pT1a | rhTSH | --------- | 3700 | LN (III) | nUS | 9 mm | ------------------ | ---------------- | 0.79 | 0.42 | 103.1 | 1.00 |
| **19** | 47 | M | LL | PTC | pT1a | rhTSH | --------- | 3700 | LN (VI) | nUS | 6 mm | ------------------ | ---------------- | 0.14 | 0.14 | 95.3 | 0.14 |
| **20** | 59 | M | RL | PTC | pT1a | rhTSH | --------- | 2220 | LN (III) | nUS | 5 mm | ------------------ | ---------------- | 0.18 | 0.14 | 42.7 | 0.14 |
| **21** | 76 | M | LL | PTC | pT1b | rhTSH | --------- | 2220 | LN (I) | nUS | 6 mm | ------------------ | ---------------- | 0.10 | 0.14 | 53.8 | 0.60 |
| **22** | 68 | M | LL | PTC | pT2 | rhTSH | --------- | 2220 | LN (VI) | nUS | 8 mm | ------------------ | ---------------- | 0.02 | 0.14 | 44.0 | 0.14 |
| **23** | 35 | F | B | PTC | pT1b(m) | rhTSH | --------- | 2220 | LN (III) | nUS | 9 mm | performed(*) | ---------------- | 0.56 | 0.14 | 134.0 | 10.5 |
| **24** | 35 | F | LL | PTC | pT1a | rhTSH | --------- | 3700 | LN (III-VI) | nUS | 11 mm | performed(*) | performed(#) | 0.01 | 0.14 | 101.8 | 14.4 |
| **25** | 37 | F | LL | PTC | pT1a | rhTSH | --------- | 4588 | LN (VII) | nUS-CT | 12 mm | ------------------ | ---------------- | 0.02 | 0.42 | 92.2 | 13.4 |
| **26** | 44 | F | LL | PTC | pT1a | rhTSH | --------- | 3700 | LN (VII) | nUs-CT | 13 mm | ------------------ | ---------------- | 0.57 | 0.14 | 119.7 | 15.0 |
| **27** | 49 | F | LL | PTC | pT1b | rhTSH | --------- | 3700 | L | CT | --------- | ------------------ | ---------------- | 0.04 | 0.26 | 196.0 | 35.3 |
| **28** | 70 | F | LL | FTC | pT3 | rhTSH | --------- | 3700 | LN (VII) | nUS-CT-^18^F-FDG | 13 mm | ------------------ | ---------------- | 0.01 | 0.71 | 192.0 | 12.3 |
| **29** | 82 | F | LL | FTC | pT3m | rhTSH | --------- | 9250 | L+B | CT-^18^F-FDG | --------- | ------------------ | ---------------- | 0.10 | 4700 | 194.0 | >4700 |
| **30** | 62 | M | LL | PTC | pT1a | rhTSH | --------- | 3700 | LN (VI-IV) | nUS | 12 mm | performed(*) | performed(#) | 0.00 | 8.50 | 72.7 | 45.0 |
| **31** | 70 | M | RL | FTC | pT3 | rhTSH | --------- | 2220 | LN (VII) + L | nUS-CT-^18^F-FDG | 10 mm | ------------------ | ---------------- | 0.08 | 0.14 | 74.7 | 36.0 |
| **32**  **33** | 68  25 | M  F | B  B | PTC  PTC | pT1a  pT1a(m) | rhTSH  THW | ---------  4 | 3700  3700 | LN (III)  LN (III) | nUS  nUS | 8mm  6 mm | performed(*)  ------------------ | ----------------  ---------------- | 0.60  0.20 | 0.14  0.14 | 125  53.8 | 0.14  3.40 |
| **34** | 30 | F | RL | PTC | pT1b | THW | 2.6 | 2753 | LN (III) | nUS | 6 mm | ------------------ | ---------------- | 0.20 | 0.14 | 65.9 | 1.00 |
| **35** | 31 | F | B | PTC | pT1b(m) | THW | 3.7 | 2753 | LN (VI) | nUS | 7 mm | ------------------ | ---------------- | 0.20 | 0.14 | 65.5 | 7.10 |
| **36** | 33 | F | B | PTC | pT1b(m) | THW | 4 | 2220 | LN (VI) | nUS | 6 mm | ------------------ | ---------------- | 0.40 | 0.14 | 131.0 | 0.14 |
| **37** | 37 | F | LL | PTC | pT3 | THW | 7.3 | 2753 | LN (VI) | nUS | 8 mm | ------------------ | ---------------- | 0.00 | 0.14 | 99.0 | 0.14 |
| **38** | 41 | F | LL | PTC | pT1a | THW | 21 | 3700 | LN (VI) | nUS | 11 mm | ------------------ | performed(#) | 0.10 | 0.14 | 33.3 | 8.46 |
| **39** | 43 | F | RL | PTC | pT3 | THW | 10.5 | 3700 | LN (IV) | nUS | 10 mm | performed(*) | performed(#) | 0.01 | 0.14 | 134.0 | 0.64 |
| **40** | 43 | F | I | PTC | pT1b | THW | 5 | 3700 | LN (VI) | nUS | 7 mm | ------------------ | ---------------- | 0.04 | 0.14 | 90.0 | 0.14 |
| **41** | 45 | F | B | PTC | pT1a(m) | THW | 2 | 2220 | LN (IV) | nUS | 13 mm | performed(*) | performed(#) | 0.78 | 0.14 | 120.0 | 2.50 |
| **42** | 47 | F | RL | PTC | pT1a | THW | 3.5 | 4558 | LN (VII) | nUs-TC | 8 mm | ------------------ | ---------------- | 0.07 | 0.14 | 67.0 | 0.14 |
| **43** | 51 | F | LL | PTC | pT3 | THW | 5.8 | 2753 | LN (III) | nUS | 7 mm | ------------------ | ---------------- | 0.01 | 0.14 | 145.0 | 0.14 |
| **44** | 52 | F | I | PTC | pT1a | THW | 9 | 3700 | LN (VI) | nUS | 11 mm | ------------------ | ---------------- | 0.19 | 0.60 | 80.0 | 2.20 |
| **45** | 55 | F | LL | PTC | pT3 | THW | 11 | 3700 | LN (VI) | nUS | 13 mm | ------------------ | performed(#) | 0.13 | 0.14 | 33.6 | 8.40 |
| **46** | 58 | F | I | PTC | pT1a | THW | 2.5 | 2753 | LN (VI) | nUS | 10 mm | ------------------ | ---------------- | 0.74 | 0.14 | 70.0 | 1.00 |
| **47** | 59 | F | B | PTC | pT3m | THW | 10 | 3700 | LN (VI) | nUS | 9 mm | ------------------ | ---------------- | 0.20 | 0.14 | 37.8 | 3.60 |
| **48** | 62 | F | RL | PTC | pT1b | THW | 12 | 2753 | LN (III) | nUS | 9 mm | performed(*) | ---------------- | 0.79 | 0.14 | 55.8 | 2.90 |
| **49** | 64 | F | RL | PTC | pT1b | THW | 2 | 2205 | LN (VII) | nUs-CT | 12 mm | ------------------ | ---------------- | 0.20 | 0.14 | 100.0 | 0.14 |
| **50** | 68 | F | I | PTC | pT1b | THW | 4.2 | 3700 | LN (VII) + L | nUS-CT | 7 mm | ------------------ | ---------------- | 0.01 | 0.14 | 35.0 | 0.14 |
| **51** | 68 | F | RL | PTC | pT1b | THW | 2 | 1110 | LN (VI) | nUS | 7 mm | ------------------ | ---------------- | 0.02 | 0.14 | 51.0 | 0.14 |
| **52** | 68 | F | RL | FTC | pT2 | THW | 4 | 4588 | LN (VI) | nUS | 5 mm | ------------------ | ---------------- | 0.02 | 0.14 | 39.4 | 0.14 |
| **53** | 68 | F | LL | PTC | pT1b | THW | 6 | 4588 | LN (III) | nUS | 6 mm | ------------------ | ---------------- | 0.13 | 0.14 | 71.3 | 0.14 |
| **54** | 69 | F | LL | PTC | pT1a | THW | 6.9 | 2753 | LN (III) | nUS | 8mm | performed(*) | ---------------- | 0.40 | 0.14 | 63.6 | 0.14 |
| **55** | 69 | F | I | PTC | pT1a | THW | 8 | 4588 | LN (VII) | nUs-CT | 11 mm | ------------------ | ---------------- | 0.05 | 0.14 | 44.8 | 1.00 |
| **56** | 73 | F | RL | PTC | pT1b | THW | 9 | 4588 | LN (VI) | nUS | 6 mm | ------------------ | ---------------- | 0.06 | 0.14 | 34.2 | 0.77 |
| **57** | 25 | M | LL | PTC | pT1a | THW | 6 | 3700 | LN (VI) | nUS | 7 mm | ------------------ | ---------------- | 0.92 | 0.14 | 221.0 | 0.83 |
| **58** | 39 | M | LL | PTC | pT1bm | THW | 17 | 3700 | LN (VII) | nUs-CT | 13 mm | ------------------ | ---------------- | 0.20 | 0.14 | 80.7 | 7.50 |
| **59** | 42 | M | RL | PTC | pT1b | THW | 6 | 4210 | LN (IV) | nUS | 11 mm | performed(*) | performed(#) | 0.20 | 0.14 | 40.6 | 5.10 |
| **60** | 43 | M | B | PTC | pT3m | THW | 17 | 2526 | LN (VII) | nUs-MR | 12 mm | ------------------ | ---------------- | 0.20 | 0.14 | 77.6 | 8.00 |
| **61** | 44 | M | LL | PTC | pT1a | THW | 4.8 | 3700 | LN (III) | nUS | 10 mm | performed(*) | performed(#) | 0.01 | 0.14 | 62.3 | 0.14 |
| **62** | 49 | M | B | PTC | pT3m | THW | 5 | 4588 | LN (VI) | nUS | 6 mm | ------------------ | ---------------- | 0.40 | 0.14 | 198.0 | 6.50 |
| **63** | 51 | M | B | PTC | pT3m | THW | 7.8 | 4588 | LN (II) | nUS | 10 mm | performed(*) | ---------------- | 0.28 | 0.39 | 85.0 | 4.20 |
| **64** | 54 | M | B | PTC | pT1a(m) | THW | 12 | 3700 | LN (IV) | nUS | 10 mm | performed(*) | performed(#) | 0.90 | 0.14 | 50.0 | 3.40 |
| **65** | 35 | F | B | PTC | pT3m | THW | 2 | 4588 | LN (III-IV) | nUS | 10 mm | performed(*) | performed(#) | 0.90 | 0.14 | 74.0 | 36.0 |
| **66** | 37 | F | LL | PTC | pT3 | THW | 7.5 | 2509 | LN (III-V) | nUS | 10 mm | performed(*) | performed(#) | 0.19 | 4.39 | 89.0 | 168.0 |
| **67** | 44 | F | RL | PTC | pT3 | THW | 7 | 3700 | LN (VI) | nUS | 10 mm | ------------------ | ---------------- | 0.60 | 2.54 | 71.0 | 40.8 |
| **68** | 47 | F | RL | PTC | pT3 | THW | 6.4 | 2220 | LN (III) | nUS | 9 mm | performed(*) | performed(#) | 0.10 | 0.14 | 50.0 | 14.3 |
| **69** | 47 | F | LL | FTC | pT1b | THW | 1.70 | 3700 | B | TC-^18^F-FDG | --------- | ------------------ | ---------------- | 0.20 | 0.14 | 36.1 | 2023 |
| **70** | 48 | F | RL | FTC | pT3 | THW | 5.6 | 3700 | B | TC-^18^F-FDG | --------- | ------------------ | ---------------- | 0.20 | 0.14 | 104.0 | 114.0 |
| **71** | 49 | F | RL | PTC | pT1a | THW | 3 | 2753 | LN (III-V) | nUS | 9 mm | ------------------ | ---------------- | 0.10 | 4.00 | 88.0 | 60.7 |
| **72** | 50 | F | B | PTC | PT3m | THW | 7 | 6882 | LN (VII) | nUs-CT | 12 mm | ------------------ | ---------------- | 0.06 | 2.54 | 71.0 | 40.8 |
| **73** | 52 | F | RL | PTC | pT1a | THW | 29 | 3700 | LN (IV) | nUs-CT | 11 mm | performed(*) | performed(#) | 0.02 | 3.70 | 36.6 | 86.1 |
| **74** | 54 | F | RL | PTC | pT1a | THW | 22 | 2753 | LN (II-III) | nUs-CT | 10 mm | performed(*) | ---------------- | 0.26 | 0.14 | 99.0 | 30.6 |
| **75** | 55 | F | LL | PTC | pT1b | THW | 9.7 | 4588 | LN (VII) | nUs-CT | 14 mm | ------------------ | ---------------- | 0.04 | 0.14 | 52.2 | 10.0 |
| **76** | 59 | F | RL | PTC | pT1a | THW | 18 | 3700 | LN (VII) | nUs-CT | 14 mm | ------------------ | ---------------- | 0.23 | 0.14 | 73.0 | 12.3 |
| **77** | 62 | F | LL | FTC | pT3 | THW | 7.7 | 4588 | L | TC-^18^F-FDG | --------- | ------------------ | ---------------- | 0.05 | 102 | 90.0 | 160.0 |
| **78** | 69 | F | I | PTC | pT1a | THW | 4.5 | 4588 | LN (VII) | nUs-CT-MR | 12 mm | ------------------ | ---------------- | 0.04 | 2.00 | 30.8 | 39.0 |
| **79** | 35 | M | B | PTC | pT2m | THW | 4.60 | 2753 | LN (III) | nUs | 8 mm | ------------------ | ---------------- | 0.58 | 18.7 | 80.5 | 161.0 |
| **80** | 56 | M | I | PTC | pT2m | THW | 15 | 3700 | LN (VII) | nUs-CT-MR | 10 mm | ------------------ | ---------------- | 0.30 | 0.14 | 31.0 | 20.8 |
| **81** | 70 | M | LL | PTC | pT3 | THW | 19.7 | 1636 | LN (VI-VII) | nUs-CT-MR | 12 mm | ------------------ | ---------------- | 0.20 | 0.14 | 33.1 | 13.5 |
| **82** | 73 | M | B | PTC | pT3m | THW | 11.2 | 3700 | LN (VII) | nUS-CT | 8 mm | ------------------ | ---------------- | 0.84 | 0.14 | 44.1 | 0.14 |
