# Supplementary material for: Undetectable or low (<1 ng/ml) postsurgical thyroglobulin values do not rule out metastases in early stage differentiated thyroid cancer patients
Source: Oncotarget. 2018 Apr 3;9(25):17491–500. doi: 10.18632/oncotarget.24766 (PMC5915131; doi:10.18632/oncotarget.24766)
Supplement: Supplementary file 3 [file oncotarget-09-17491-s003.docx]

**Supplementary Table 2: Demographic, clinical, pathological and scintigraphic data of 22 pT1a patients with a-Tg <1 ng/ml and metastasis(es) at post-therapeutic imaging**

| **Patient Number** | **Age** | **Sex** | **Tumor site** | **Histology** | **pT-staging** | **Stimulation**  **strategy** | **RAIU**  **(%)** | **Activity (MBq)** | **Metastases topography on post-therapeutic imaging** | **Targeted Diagnostic studies** | **Lymph-nodes size** | **Node biopsy** | **Lympho-adenectomy** | **TSH**  **on L-T4**  **(uIU/ml)** | **ps-Tg**  **(ng/ml)** | **TSH**  **at TRA**  **(uIU/ml)** | **Tg**  **at TRA**  **(ng/ml)** |
| --- | --- | --- | --- | --- | --- | --- | --- | --- | --- | --- | --- | --- | --- | --- | --- | --- | --- |
|  |  |  |  |  |  |  |  |  |  |  |  |  |  |  |  |  |  |
| **1** | 26 | F | RL | PTC | pT1a | rhTSH | --------- | 4588 | LN (VI) | nUS | 7 mm | ------------------ | ---------------- | 0.03 | 0.24 | 75.2 | 0.35 |
| **2** | 34 | F | B | PTC | pT1a(m) | rhTSH | --------- | 2220 | LN (II) | nUS | 7 mm | ------------------ | ---------------- | 0.15 | 0.14 | 115.0 | 0.14 |
| **3** | 40 | F | RL | PTC | pT1a | rhTSH | --------- | 4588 | LN (VII) | nUS-TC | 9 mm | ------------------ | ---------------- | 0.17 | 0.14 | 83.0 | 0.14 |
| **4** | 40 | F | LL | PTC | pT1a | rhTSH | --------- | 4588 | LN (VI) | nUS | 7 mm | ------------------ | ---------------- | 0.46 | 0.14 | 56.6 | 0.14 |
| **5** | 48 | F | B | PTC | pT1a(m) | rhTSH | --------- | 3700 | LN (VI) | nUs | 6 mm | ------------------ | ---------------- | 0.45 | 0.14 | 63.1 | 0.14 |
| **6** | 48 | F | B | PTC | pT1a(m) | rhTSH | --------- | 2948 | LN (I) | nUS | 6 mm | ------------------ | ---------------- | 0.01 | 0.14 | 145.9 | 0.14 |
| **7** | 53 | F | LL | PTC | pT1a | rhTSH | --------- | 2220 | LN (II) | nUS | 11 mm | performed(*) | performed(#) | 0.09 | 0.49 | 128.0 | 0.68 |
| **8** | 59 | F | RL | PTC | pT1a | rhTSH | --------- | 2220 | LN (IV) | nUS | 6 mm | ------------------ | ---------------- | 0.09 | 0.14 | 120.0 | 0.62 |
| **9** | 68 | F | RL | PTC | pT1a | rhTSH | --------- | 3700 | LN (III) | nUS | 6 mm | ------------------ | ---------------- | 0.02 | 0.14 | 54.8 | 0.27 |
| **10** | 69 | F | LL | PTC | pT1a | rhTSH | --------- | 3700 | LN (II) | nUS | 9 mm | performed(*) | performed(#) | 0,00 | 0.14 | 141.0 | 0.31 |
| **11** | 25 | M | I | PTC | pT1a | rhTSH | --------- | 3700 | LN (VII) | nUS-CT-MR | 5 mm | ------------------ | ---------------- | 0.04 | 0.23 | 74.4 | 0.52 |
| **12** | 31 | M | B | PTC | pT1a(m) | rhTSH | --------- | 4588 | LN (III) | nUS | 7 mm | ------------------ | ---------------- | 0.42 | 0.14 | 88.8 | 0.14 |
| **13** | 43 | M | RL | PTC | pT1a | rhTSH | --------- | 3700 | LN (III) | nUS | 9 mm | ------------------ | ---------------- | 0.79 | 0.42 | 103.1 | 1.00 |
| **14** | 47 | M | LL | PTC | pT1a | rhTSH | --------- | 3700 | LN (VI) | nUS | 6 mm | ------------------ | ---------------- | 0.14 | 0.14 | 95.3 | 0.14 |
| **15** | 59 | M | RL | PTC | pT1a | rhTSH | --------- | 2220 | LN (III) | nUS | 5 mm | ------------------ | ---------------- | 0.18 | 0.14 | 42.7 | 0.14 |
| **16** | 68 | M | B | PTC | pT1a | rhTSH | --------- | 3700 | LN (III) | nUS | 8mm | performed(*) | ---------------- | 0.60 | 0.14 | 125 | 0.14 |
| **17** | 47 | F | RL | PTC | pT1a | THW | 3.5 | 4558 | LN (VII) | nUs-TC | 8 mm | ------------------ | ---------------- | 0.07 | 0.14 | 67.0 | 0.14 |
| **18** | 58 | F | I | PTC | pT1a | THW | 2.5 | 2753 | LN (VI) | nUS | 10 mm | ------------------ | ---------------- | 0.74 | 0.14 | 70.0 | 1.00 |
| **19** | 69 | F | LL | PTC | pT1a | THW | 6.9 | 2753 | LN (III) | nUS | 8mm | performed(*) | ---------------- | 0.40 | 0.14 | 63.6 | 0.14 |
| **20** | 69 | F | I | PTC | pT1a | THW | 8 | 4588 | LN (VII) | nUs-CT | 11 mm | ------------------ | ---------------- | 0.05 | 0.14 | 44.8 | 1.00 |
| **21** | 25 | M | LL | PTC | pT1a | THW | 6 | 3700 | LN (VI) | nUS | 7 mm | ------------------ | ---------------- | 0.92 | 0.14 | 221.0 | 0.83 |
| **22** | 44 | M | LL | PTC | pT1a | THW | 4.8 | 3700 | LN (III) | nUS | 10 mm | performed(*) | performed(#) | 0.01 | 0.14 | 62.3 | 0.14 |

***PTC*** *:* Papillary Thyroid Cancer, ***FTC:*** Follicolar Thyroid Cancer, ***RL:*** Right Lobe, ***LL:*** Left Lobe, ***I:*** Isthmus, ***B:*** Bilateral; ***pTm:*** multifocal, ***LN: lymph-node (****Lymph-node metastases’ level according to Robbins’s classification*), ***L:*** Lung; ***B:*** Bone; ***nUS:***  neck Ultrasound; ***CT***: Computed Tomography; ***MR*:** Magnetic Resonance; *** :**Tg > 500 ng/ml; **#:** metastasis(es) confirmed by histology.
